# Comment on “Noninvasive prenatal screening at low fetal fraction: comparing whole‐genome sequencing and single‐nucleotide polymorphism methods”

**DOI:** 10.1002/pd.5072

**Published:** 2017-07-03

**Authors:** Allison Ryan, Kimberly A. Martin

**Affiliations:** ^1^ Natera Statistics San Carlos CA USA; ^2^ Natera San Carlos CA USA

This correspondence is in reference to the article entitled, ‘Noninvasive Prenatal Screening at Low Fetal Fraction: Comparing Whole‐Genome Sequencing and Single‐Nucleotide Polymorphism Methods’ by Artieri, *et al.*, which was recently published in *Prenatal Diagnosis*.^1^ The objective of the publication was to compare the performance of two non‐invasive prenatal testing (NIPT) methods, whole‐genome sequencing (WGS) and single nucleotide polymorphism (SNP)‐based analysis of cell‐free DNA, for the detection of fetal trisomies, specifically at low fetal fractions. The authors make strong clinical claims about the inferior performance of SNP‐based NIPT compared with the WGS method that are invalidated by two significant methodological flaws – (1) the WGS model does not address inherent biological and measurement variations, and (2) the model for the SNP‐based method disregards the method's ability to establish linkage between SNPs. Compounding these issues, neither simulation model was validated by showing that it produces realistic sequencing data.

To point one, the approach used for the WGS model was based on a theoretical Poisson distribution and did not incorporate any sources of variance other than random sampling of the number of reads. It is, however, widely accepted that WGS‐generated NIPT data are affected by several additional sources of variance,^2^ both biological and laboratory‐process driven, which lead to reduced performance at low fetal fraction.^3^, ^4^ These include variability in fetal DNA concentration, pre‐sequencing DNA amplification, and GC content. In the described simulation approach, any underestimate of WGS process variance would result in the trisomy classification limit being set artificially low, leading directly to improved apparent sensitivity. Furthermore, it is standard practice that simulation‐based analyses be supported by evidence that the simulation produces realistic data – in the absence of such validation, there is no justification for using the simulation to make claims about clinical performance.

To point two, the modeling of the SNP‐based classification method was incomplete. The authors' implementation of the SNP‐based classification method incorrectly assumes that each SNP's fetal genotype is inherited independently from adjacent loci, ignoring the information available using linkage. In contrast, Natera's SNP‐based method incorporates the effect of chromosome recombinations and homolog inheritance (i.e. linkage) that are critical to accurately detecting trisomies. As it is missing this critical component, the SNP‐based classifier implemented by the authors cannot be used to predict performance limitations of a fully implemented method.

Finally, high sensitivity of the SNP‐based NIPT method was analytically validated using plasma from affected pregnancies.^5^, ^6^ Additionally, SNP‐based methods have the unique ability to detect triploidy,^7^ vanishing twins and molar pregnancies, which further increases their overall ability to detect aneuploidy. The article by Artieri, *et al.* does not provide sound justification for its claims regarding poor sensitivity of the SNP method, nor has Counsyl published validation data demonstrating clinical sensitivity of their own WGS‐based test.^1^
